# Oxygen Distributions—Evaluation of Computational Methods, Using a Stochastic Model for Large Tumour Vasculature, to Elucidate the Importance of Considering a Complete Vascular Network

**DOI:** 10.1371/journal.pone.0166251

**Published:** 2016-11-18

**Authors:** Jakob H. Lagerlöf, Peter Bernhardt

**Affiliations:** Department of Radiation Physics, University of Gothenburg, Gothenburg, Sweden; Universita degli Studi di Catania, ITALY

## Abstract

**Purpose:**

To develop a general model that utilises a stochastic method to generate a vessel tree based on experimental data, and an associated irregular, macroscopic tumour. These will be used to evaluate two different methods for computing oxygen distribution.

**Methods:**

A vessel tree structure, and an associated tumour of 127 cm^3^, were generated, using a stochastic method and Bresenham’s line algorithm to develop trees on two different scales and fusing them together. The vessel dimensions were adjusted through convolution and thresholding and each vessel voxel was assigned an oxygen value. Diffusion and consumption were modelled using a Green’s function approach together with Michaelis-Menten kinetics. The computations were performed using a combined tree method (CTM) and an individual tree method (ITM). Five tumour sub-sections were compared, to evaluate the methods.

**Results:**

The oxygen distributions of the same tissue samples, using different methods of computation, were considerably less similar (root mean square deviation, RMSD≈0.02) than the distributions of different samples using CTM (0.001< RMSD<0.01). The deviations of ITM from CTM increase with lower oxygen values, resulting in ITM severely underestimating the level of hypoxia in the tumour. Kolmogorov Smirnov (KS) tests showed that millimetre-scale samples may not represent the whole.

**Conclusions:**

The stochastic model managed to capture the heterogeneous nature of hypoxic fractions and, even though the simplified computation did not considerably alter the oxygen distribution, it leads to an evident underestimation of tumour hypoxia, and thereby radioresistance. For a trustworthy computation of tumour oxygenation, the interaction between adjacent microvessel trees must not be neglected, why evaluation should be made using high resolution and the CTM, applied to the entire tumour.

## Introduction

A sufficient tumour vasculature is crucial for tumour cell division and for tumour growth. The tumour is able to interfere with its vasculature, through angiogenesis, in order to increase the oxygen delivery to the cells as the tumour volume expands and the distance between blood vessels increases. Depending on the geometry and size of a tumour and the structure of its vasculature, the development of the tumour as well as the feasibility of successful treatment may differ substantially.[1,2] Consequently, knowledge of the vascular architecture, of tumours in general and of individual tumours in particular, is valuable as it may be a determining factor to whether the tumour is eradicated or not.

Over the years, many attempts have been made to map and model the behaviour of tumour vasculature. Starting with the Krogh cylinder in 1919, which may be considered the foundation of vascular modelling,[3] models have developed through countless two (2D) and three dimensional (3D) approaches of various complexity and applicability. Recent examples include single cell based 2D models,[4] 3D models with constant vessel oxygen level,[5] numerical models using greens functions[6] and models including heterogeneity of vascular oxygen content.[7,8]

With the progress of medical imaging techniques and improved computational capacity, the amount of available information has expanded rapidly and models become increasingly sophisticated and realistic. For example, the work by Adhikarla and Jeraj,[5] where they build a semi-stochastic vessel tree model by combining imaged macrovessel with two generations of simulated vessels. They adapted the density of larger simulated vessels to a measured oxygen map, while using a branching statistics, resembling the measurements by Op Den Buijs et.al,[9] to develop the capillary network. Due to computer limitations, they were not able to properly evaluate the model at a high resolution, but managed to, in high-resolved sub volumes, achieve good agreement between simulations and the oxygen map, using constant pO_2_ of 60 mmHg in the vessels. These results are encouraging for further development of purely stochastic microscopic vessel models in large scale.

In the present state of the art, vascular models are 3D with non-parallel vessels, altering intra-vascular oxygen content and supply-dependent consumption in the tissue.[8] Because of the complexity of the vasculature, numerical methods are commonly used, which essentially correlates model dimensions, resolution and accuracy to runtime and computational workload. Obviously limitations apply, regardless of model type and design. Models of today typically handle computations on tissue dimensions on the order of millimetres, with a decent resolution. Beyond this size, assumptions of symmetry or repetition are employed.[6,7] However, for radiologically observable clinical tumours such approach will only represent a sub-volume of the tumour. Therefore, this approach will not embrace macroscopic variations in the vascular fractions and its impact on the spatial oxygen distribution in a radiologically observable tumour size. With increasing precision in external radiation treatment, such as biologically guided dose painting comes a need for detailed knowledge of tumour radio resistance and hypoxia on a microscopic as well as a macroscopic level.

The aim of this study is to construct a schematic tumour model that catches both the macroscopic and microscopic vessel and oxygen heterogeneity within a clinically relevant tumour size. For this purpose, we will utilise a stochastic method for the creation of a large, steady-state vessel tree, which will fill a macroscopic tumour of the size just above 100 cm^3^. This tumour model will be used to compare the macroscopic variations of the oxygen distributions in millimetre sub-volumes in order to evaluate the ability to detect hypoxic regions, using a simplified procedure for simulating of the oxygen distribution, the individual tree method (ITM). The simplicity of this method makes it possible to apply it to the entire tumour model. As baseline, results from a highly resolved more demanding greens function and Michaelis-Menten approach, the combined tree method (CTM), is used. Evaluations are made for five randomly selected sub-volumes.

Further, the similarity of the different sub-volumes of the tumour model, using the output from the advanced model, is investigated in order to determine whether the highly resolved millimetre-scale samples may be used to approximate the entire tumour oxygen distribution.

## Methods

To effectively generate the entire vessel architecture in a macroscopic tumour and from this vessel arrangement compute the oxygen distribution, we first developed a non-permeable macrovessel tree to which permeable microvessel trees were attached.[10] One microvessel tree was attached to each leaf node of the macrovessel trees to make a continuous transition from lower generation vessels to higher generation vessels. The macrovessel trees included nine generations, which made the end branches about 100 μm wide, using vessel dimension data (segment lengths and radii) from Debbaut et al.[11] Vessels smaller than this, were considered permeable[12] vessels and the dimensions were calculated using the trend lines from their data fits. [11] Five microvessel generations, down to an average segment length of 50 μm (including variations, some vessel segments were as short as 40 μm) were included. Both the work of Debbaut et al.[11] and of Op Den Buijs et al.[9] describe the vessel structure of liver vasculature. We used this data because the liver vessels are undisturbed by anatomic structures, which allows them to develop without interference and therefore makes the liver suitable for studies of general vasculature, adjusting vessel densities of course, since the liver vasculature is rather dense. [9] Macro- and microvessel trees were generated using a stochastic method. The trees and tumour tissue were assumed to appear instantly and were time invariant, why the simulation results are steady-state.

Oxygen transport and consumption, involving only microvessels, were computed. The spatial resolution was 10 μm, unless otherwise stated. All computations were made using a portable workstation, with Intel® Core™ i7 processor and 16 GB RAM, running 64-bit MATLAB® R2014b. The parameter setting, used in the computations, is displayed in Table 1.

**Table 1:** Symbols and dimensions of quantities and parameter setting used in the computations.

Quantity	Symbol	Value	Dimension
Concentration (probability of finding a molecule)	*c*	-	μm^-3^
Oxygen consumption rate	*C*	-	mmHg s^-1^
Oxygen demand at unlimited supply[6]	*C*_*0*_	15	mmHg s^-1^
Diffusion coefficient for oxygen in tissue[6]	*D*_*O*_	2000	μm^2^s^-1^
Hypoxic fractions (threshold 0.01 mmHg)	*HF*_*0*.*01*_	-	-
Hypoxic fractions (threshold 1 mmHg)	*HF*_*1*_	-	-
Hypoxic fractions (threshold 5 mmHg)	*HF*_*5*_	-	-
Normalisation constant[13]	*k*	1000∙(4∙*D*_*O*_∙*t*∙π)^-3/2^	μm^-3^s^3/2^
Michaelis constant (*pO*_*2*_ at which *C* = *C*_*0*_/2)[14]	*K*_*M*_	1	mmHg
Oxygen partial pressure	*pO*_*2*_	-	mmHg
Distance	*r*	-	μm
Time step	*t*	0.1	s
Azimuthal angle	ϕ	-	-
Polar angle	θ	-	-

### Generation of macrovessel trees

**Fig 1:**
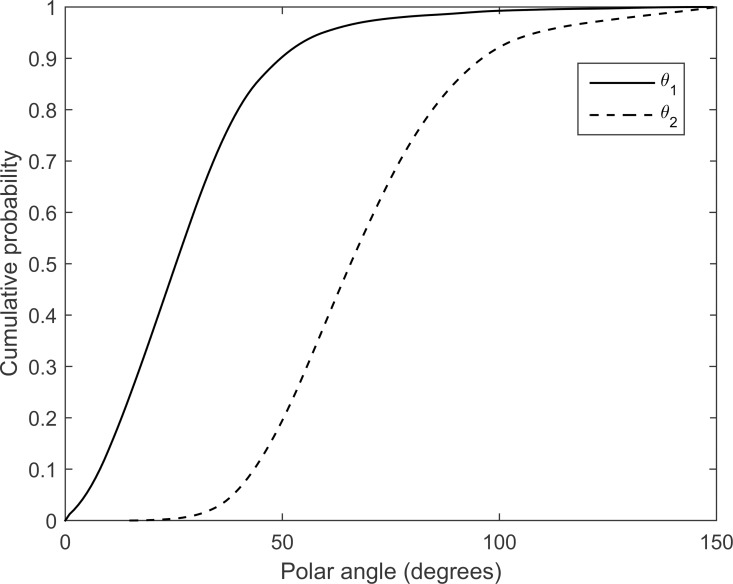
Angular distribution. The cumulative distribution function of the polar angles, according to Op Den Buijs et al., for the two branches.[9].

Using experimental vessel data for branching characteristics,[9] fully five hundred macrovessel trees were included in the model, to roughly meet a functional microvessel density target of 0.04, given an average total volume per microvessel tree of 0.039 mm^3^.[7,15] From a random starting point, distances and directions (azimuthal angle, ϕ and polar angle, θ) were sampled from respective distribution, using the inverse transform method,[16] determining the coordinates of the following bifurcations. The distribution of azimuthal angles is uniform and the cumulative distributions of the polar angles, according to Op Den Buijs et al., for the two branches are displayed in Fig 1.[9]

**Fig 2:**
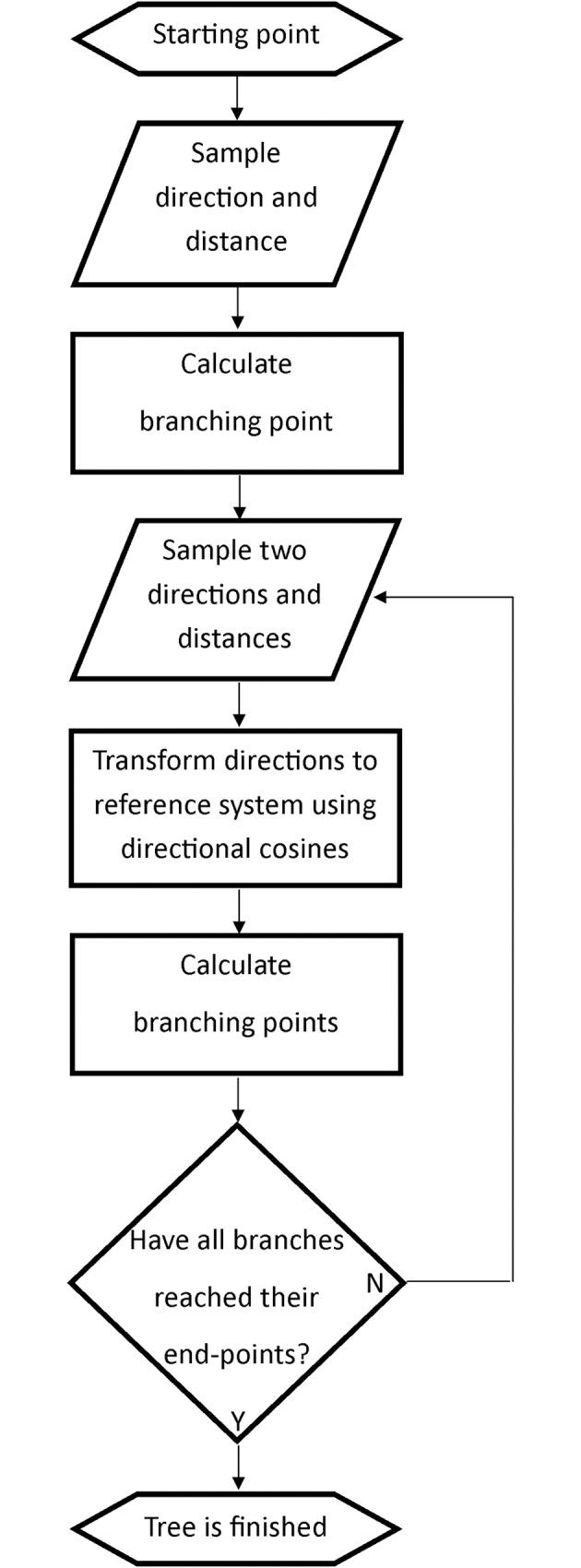
Generation of vessel trees. The process of stochastic generation of vessel trees. New directions and distances were sampled iteratively until all branches had reached the predetermined vessel generation number.

From every branching point, two narrower sub-vessels emerged in different directions, following the same principle. For each generation, the vessel radius decreased by 5–15 μm.[11] Each new direction was relative to the previous and consequently, for the preservation of a universal frame of reference, had to be converted using direction cosines.[17] The sampling process was repeated, for each sub-vessel to develop a complete tree with nine vessel generations. In this case, only the leaf node coordinates of each branch were stored. These coordinates were used as starting points for the microvessel trees. The general principle of vessel tree generation is shown in a flow chart (Fig 2).

The coordinates of the leaf nodes of all branches were stored and a tumour perimeter was defined as the surface containing all points, calculated by triangulation. In this case, the tumour volume is 127 cm^3^.

### Generation of microvessel trees and oxygen sub-distributions

Using the assumptions and method described above, together with vessel dimension and prevalence data,[11] one hundred separate microvessel trees were simulated, including another five vessel generations. These trees included vessel segment lengths from about 400 down to 40 μm. Vessels smaller than this were discarded due to finite resolution. In this case, an actual tree was generated through connection of the branching points using Bresenham’s line algorithm, to optimise the discrete approximation of a continuous line and avoid discontinuities in the voxel representation.[18] Each vessel segment was convolved with a spherical smoothing kernel, to set the vessel diameter according to experimental findings [11] and thresholded to define the vessel surface. The microvessel tree was stored in a 3D matrix.

An oxygen level was assigned to every vessel voxel, starting at 100 mmHg (arterial oxygen level) at the emerging point (the leaf node of the parent macrovessel tree) and falling off linearly with distance from this point, to a minimum value of 40 mmHg (venous oxygen level) in the most distant voxel, i.e. the leaf node of the longest trajectory in each microvessel tree. These values were chosen because they roughly span the oxygen tension interval of functional blood vessels.[19] Every leaf node in the macrovessel tree was randomly associated with one of the microvessel trees (overlapping leaf nodes were removed). In total, 130955 microvessel trees were used for this tumour.

The oxygen matrices for each microvessel tree were temporarily padded with zeros to avoid edge effects. Oxygen diffusion was computed, for each of the 100 microvessel tree types, through repeated convolution of the oxygen matrix, for the respective tree, with a Gaussian kernel,[13,16] since the Gaussian (Eq 1) is the Green’s function of the diffusion equation.

$$c(r,t)={k∙t}^{\frac{-3}{2}}∙e^{\frac{-r^{2}}{4D_{0}∙t}}$$ (1)

Between iterations, tissue oxygen consumption was calculated, and subtracted, using Michaelis-Menten kinetics (Eq 2). [14]

$$C=\frac{C_{0∙{pO}_{2}}}{{pO}_{2}+K_{M}}$$ (2)

Iterations continued until the oxygen field volume reached a steady-state. These oxygen distributions were used by the ITM

### Tumour oxygen distribution computations

Different ways of determining the oxygen distribution in the entire tumour were compared. The sub-distributions determined in section 2 B may not be summed up into a macroscopic distribution, as this would discard the overlapping voxels, that most likely exist at this vessel density, resulting in an over-estimation of the oxygenation. Consequently, the geometric information needs to be preserved. The preferred method would be to inscribe all 130955 microvessel trees in the tumour, using a resolution of 10 μm, sufficient to depict the vessels properly. The oxygen distribution of the tumour could then be determined using the iterative convolution method described in section 2 B. This would require a 3D matrix of approximately 10^12^ elements, which would have to be sectioned to fit into the computer memory. The computation would take several months. To shorten the computation time, the options were to either examine the tumour partially, using the CTM or increase the voxel size and use the ITM.

### Combined Tree Method

For partial tumour examination with maintained resolution, five separate sub-volumes (3x3x3 mm^3^ each, including about 30–40 microvessel trees) of the tumour were selected and the oxygen tension was computed iteratively, using Eqs 1 and 2. The actual samples were initially padded with additional tumour voxels (these were removed after oxygen computation) in order to avoid the edge effects introduced by repeated convolutions. The resolution was 10 μm and thus, each sample consisted of 2.7∙10^7^ voxels. Because this computation method included the interaction between the separate microvessel trees, it is referred to as the CTM. These computations of the oxygen distributions for these sub-volumes were used as baseline for the ITM results.

### Individual Tree Method

For complete tumour examination, using the ITM, with increased voxel size, the entire tumour volume was down-sampled and inscribed in a matrix with a 100 μm grid, on the order of 10^9^ elements. The position of each voxel of the matrix was translated to the positions of the predetermined oxygen fields (Section 2 B) surrounding each of the microvessel trees, and the respective voxel was assigned the appropriate oxygen value. If a voxel was reached by the oxygen fields from more than one microvessel tree, the highest oxygen value was assigned to the voxel. This was done instead of summing or averaging over contributing voxels, in order to minimise the error of the oxygen content of that voxel.

The ITM approach, by necessity, failed to represent the interaction between adjacent microvessel trees which therefore needed to be treated individually. Since the coarse grid was unable to accurately represent the trees, but necessary for the entire tumour to be described at once, the sub-distributions (one for every microvessel tree) were computed at high resolution and down-sampled to fit the coarse grid. This method was able to estimate the entire tumour oxygen distribution.

### Evaluation of the oxygen computation methods

The normalised oxygen distributions of the five sample volumes were compared for CTM and ITM, through computation of hypoxic fractions for threshold levels of 0.01, 1 and 5 mm Hg and in terms of the root mean square deviation (RMSD) between the distributions of the different sample volumes. RMSD was also calculated between the distributions of each sample volume from the CTM and the corresponding volume from the ITM. These values were used to evaluate the two computation methods. To quantify the influence of the down-sampling of the sub volumes for the coarse grid used in the individual tree method, RMSD was calculated between one of the high-resolved samples and its down-sampled equivalent.

The similarity of sub-volume oxygen distributions retrieved using CTM was also investigated through pairwise two-sample Kolmogorov-Smirnov (KS) tests. This is a robust, non-parametric test, designed to compare unknown distributions. The test-statistic produced by the test is the maximum difference (D_KS_) between the cumulative distributions. The null hypothesis (H_0_) here was that the sub-volume distribution samples were drawn from the same distribution, i.e. that they were sufficiently similar to each represent the entire tumour oxygen distribution on their own. H_0_ was tested for a confidence level (α) of 0.05 and was rejected if

$$D_{KS}>c(\alpha )\sqrt{\frac{n_{1}+n_{2}}{n_{1}∙n_{2}}}$$ (3)

where *c(α)* is a parameter depending on the confidence level, e.g. *c*(0.05) = 1.36 and *c*(0.001) = 1.95, *n*_*1*_ and *n*_*2*_ are the number of values in the distributions (the sample sizes), in this case the number of voxels in the sub-volumes (2.7∙10^7^). The uncertainty of the test decreases with increasing sample size. In this context, this sample size is very large.[20]

## Results

**Fig 3:**
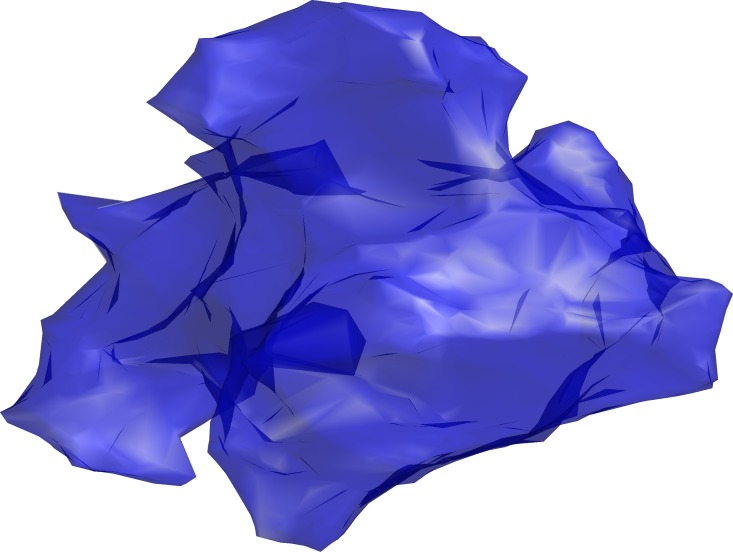
Tumour shape. The shape of the tumour generated through triangulation. The total volume of the irregular tumour is 127 cm^3^.

The method used to develop an entire vasculature in a tumour, was robust and efficient; the computation time was less than an hour once the microvessel trees were generated. This time was proportional to the number of nodes in the macrovessel tree, i.e. to the tumour size and the average vessel density. The border of the generated tumour was defined by triangulation of the positions of the most peripheral microvessel trees, which generated an irregular 127 cm^3^-sized tumour (Fig 3).

**Fig 4:**
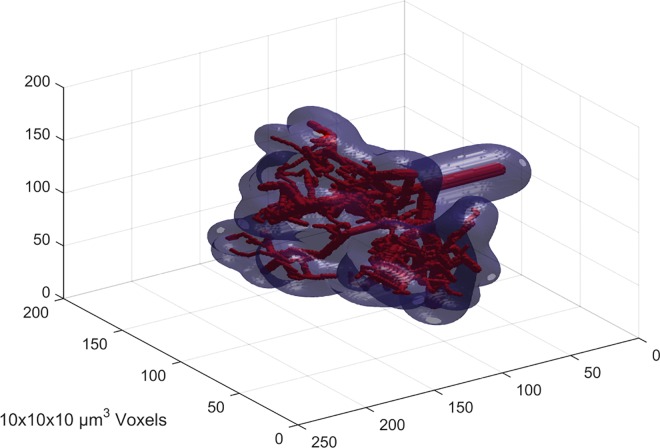
Microvessel tree. Microvessel tree (red) inside a semi-transparent isosurface (blue), marking a partial oxygen pressure level of 0.01 mmHg.

**Fig 5:**
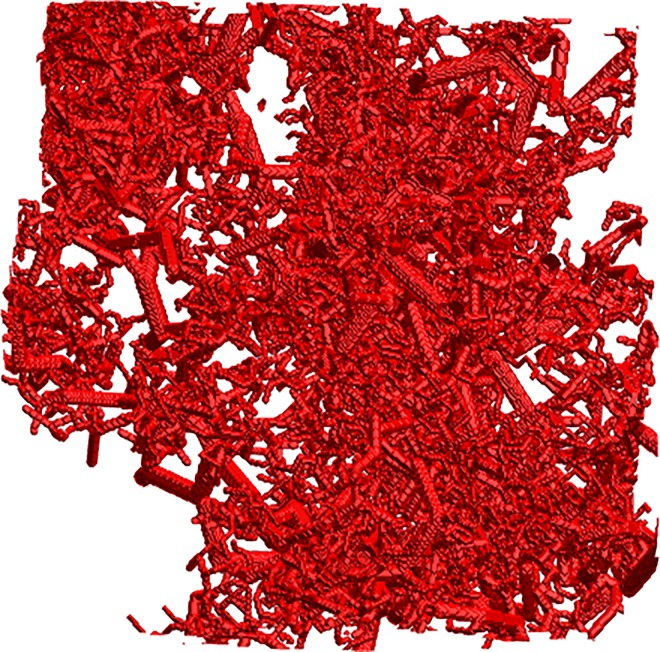
Microvascular network section. A 3x3x3 mm^3^ section of the complete microvascular network used in the combined tree method.

The computation of the oxygen distribution was quite time consuming; the computation time for the oxygen distribution in only one microvessel tree was about 15 minutes. This means that the number of individual microvessel trees used in the model to a great extent determines the computation time for the oxygen sub-distributions, while the computation times for both the ITM and CTM are independent of this. For each combined microvessel tree section of 27 mm^3^, the corresponding computation time was some 20 minutes. This time is proportional to the number of iterations, which in turn together with time-step and matrix resolution determines the accuracy of the calculation. A small time-step preserves a realistic oxygen gradient, which, in combination with small voxel size, gives more accurate calculations but requires a large number of iterations. Furthermore, the time-step should be adjusted to the voxel size for the convolution kernel to be applicable, as a high temporal resolution in combination with a low spatial dito will not allow any measureable diffusion to take place between iterations. The relation of the calculation time to the section volume (or inversely to resolution) is a bit unpredictable, mainly depending on the time for the fourier transformation of the matrix, while the section size in turn is limited by computer memory. The computation time for the whole tumour, using the ITM, was 150 hours. This time is proportional to the number of tumour voxels used, i.e. to the tumour size and the inverse of the voxel volume. One microvessel tree with corresponding isosurface at pO_2_ = 0.01 mmHg is shown in Fig 4 and a 3x3x3 mm^3^ sample of the combined tumour vasculature in Fig 5.

**Fig 6:**
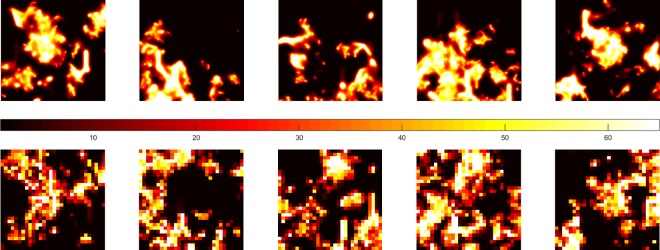
Sample slices. A 100 μm thick centre slice of samples 1 (left) through 5 (right) from the CTM (upper row) andITM (lower row) computations. The image width and height is 3 mm each and the colour bar illustrates the pO_2_ scale in mmHg.

The estimated time to compute the entire oxygen distribution in the macroscopic tumour would have been 200 days, using the CTM on the computer in question, with the current parameter setting. Therefore, we investigated if any of two more simplified approaches, i.e. the CTM applied to sub volumes or the ITM, could be used for estimating the whole tumour oxygen distribution. Fig 6 shows the oxygen tension in a section through the middle of each of the five tumour samples, where each panel represents a tissue thickness of 100 μm and an area of 9 mm^2^. The top and bottom row of each column should, in the ideal case, contain the same information. Although there are similarities, the visual impression is that the ITM overestimates the fraction of normoxic tissue.

**Fig 7:**
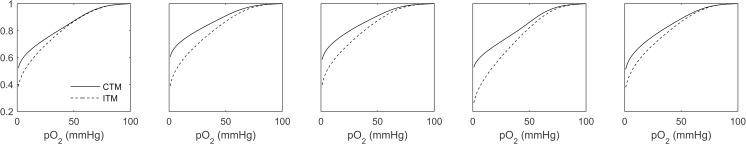
Oxygen distributions. Cumulative pO_2_ distributions for the five sample volumes. The resolution is 1 mmHg.

This impression is supported by the cumulative pO_2_ distributions of Fig 7, which show that, in all samples, the ITM gives a smaller hypoxic fraction and a larger fraction in the intermediate region, while there is little difference in the high values. From a radiosensitivity point of view, the low and intermediate pO_2_ regions are the most important, why the ITM becomes highly inappropriate.

Table 2 shows the hypoxic fractions, mean pO_2_ and the vascular fractions for the five sampled tumour segments.

**Table 2:** The vascular fractions, hypoxic fractions with threshold levels 0.01, 1 and 5 mmHg and average value and standard deviation of pO_2_for five samples from the CTM and the corresponding samples from the ITM.

Sample	HF_0.01 mmHg_	HF_1 mmHg_	HF_5 mmHg_	VF	Avg pO_2_	SD(pO_2_)
CTM 1	0.3977	0.523	0.595	0.0396	15.61	23.51
CTM 2	0.4908	0.607	0.675	0.0272	11.97	21.05
CTM 3	0.4568	0.5864	0.6577	0.0291	12.20	20.95
CTM 4	0.4380	0.5296	0.5877	0.0398	16.43	23.97
CTM 5	0.3696	0.5141	0.5964	0.0325	14.63	22.40
CTM_DS_ 1	0.4004	0.5276	0.6019	0.0396	15.35	23.19
ITM 1	0.0126	0.386	0.4829	0.0396	18.41	23.45
ITM 2	0.0086	0.3905	0.4914	0.0272	18.06	23.23
ITM 3	0.0041	0.3979	0.4980	0.0291	17.63	22.87
ITM 4	0.0013	0.2648	0.3628	0.0398	23.46	24.56
ITM 5	0.0006	0.3802	0.4792	0.0325	18.37	23.25

**Fig 8:**
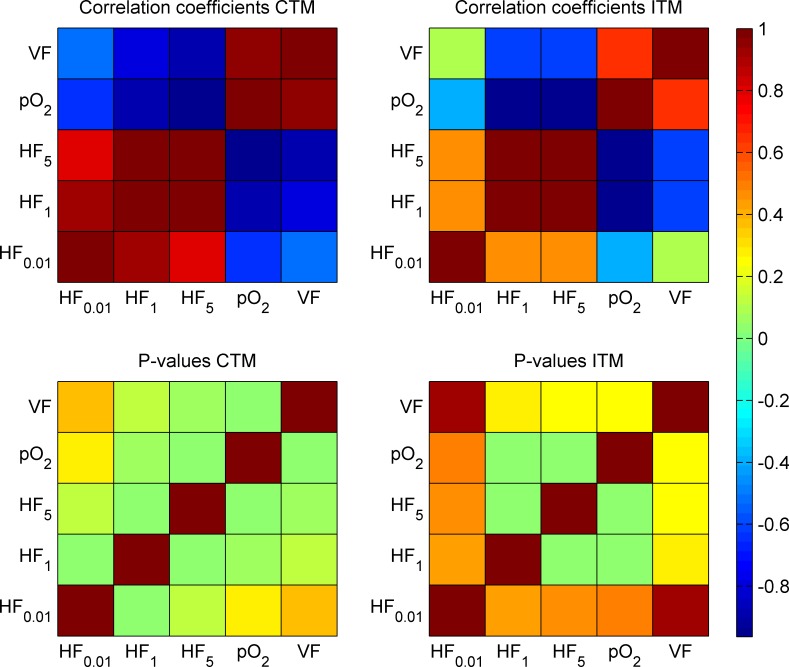
Correlation and significance. Correlation coefficients and p-values for the properties of the five samples. The correlation coefficients (upper panels) show the sign and strength of the correlations, while the p-value (lower panels) may be used to evaluate the significance. The lower left to upper right diagonals of all panels, by necessity, contain nonsense values.

There are notable differences in oxygenation between and within individual samples for both methods. For the CTM, there is, as can be expected, significant positive correlations between average pO_2_ and vascular fraction as well as significant negative correlations between HF and VF or pO_2_. The correlations are less pronounced for the lowest threshold of hypoxia. For the ITM, however, the above correlations are weak and non-significant, with the exception of the relations between HF_1_, HF_5_ and pO_2_. The correlations are quantified in terms of correlation coefficients and p-values. These are displayed in the checkerboard plots of Fig 8.

The results from the RMSD computations are shown in Table 3. When applied to normalised distributions, as in these cases, an RMSD of 0 means identical distributions and 1 means total lack of agreement between them. Each distribution from the CTM was compared against the corresponding distribution from the ITM, but also against the other distributions from the CTM. The agreement is better between independent locations within CTM than between the same location using different methods. The down-sampled version of CTM 1 is included to assure that the down-sampling itself (used in ITM) does not account for a considerable loss of information. The KS tests also clearly showed that the oxygen samples were not drawn from the same distribution, as H_0_ was rejected at α = 0.001, for all pairwise comparisons.

**Table 3:** RMSD, for five samples from the ITM and the corresponding samples from the ITM. A down-sampled version of CTM sample 1 (CTM_DS_ 1) is also included.

RMSD					
Sample	CTM 1	CTM 2	CTM 3	CTM 4	CTM 5
CTM 1	0	0.0084	0.0064	0.0011	0.0012
CTM 2	0.0084	0	0.0021	0.0078	0.0094
CTM 3	0.0064	0.0021	0	0.0058	0.0073
CTM 4	0.0011	0.0078	0.0058	0	0.0022
CTM 5	0.0012	0.0094	0.0073	0.0022	0
CTM_DS_ 1	0.0007	-	-	-	-
ITM 1	0.0139	-	-	-	-
ITM 2	-	0.0219	-	-	-
ITM 3	-	-	0.0190	-	-
ITM 4	-	-	-	0.0267	-
ITM 5	-	-	-	-	0.0135

## Discussion

Hypoxia is of interest from a prognostic point of view, not only due to its correlation with tumour aggressiveness,[21,22] but it is also important to survey for the long-known indirect effect it has been shown to have on radiation treatment outcomes. This tumour model does not attempt to accurately describe the oxygen distribution in a clinical tumour but, contrary to many simpler models, it captures the important characteristic and the heterogeneous nature of the spatial distribution of anoxia and, by extension, necrosis; unlike common general descriptions, necrotic regions often tend to distribute throughout the tumour rather than concentrate to the tumour core.[23] We know from previous work that the quantity *hypoxic fraction* is obsolete and insufficient for the prediction of radiosensitivity.[24] For the same reason, i.e. essentially loss of information due to partial volume effects, it is reasonable that the higher the resolution of the oxygen estimations, the better, at least down to the scale of one single cell. We see, in Table 2, that a down-sampling from high (voxel size 10 μm) to moderate (voxel size 100 μm) resolution causes a slight overestimation of the level of hypoxia and therefore the radioresistance. Assuming small scale heterogeneity of hypoxia and necrosis, this becomes increasingly important. The differences within and between samples, in vascular fraction and oxygenation, suggest a potential for both macro- and microscopic optimisation of absorbed dose, to improve radiation treatment results and minimise normal tissue complications.

This work further emphasises the importance of considering interaction between adjacent microvessel trees, as in the CTM. One microvessel tree is not enough to give a reliable description of the oxygenation (Table 3), even on a small scale (due to intra-tumoural variations), but considerably overestimates the normoxic fraction (Fig 7 and Table 2) and thereby would underestimate the absorbed dose required for tumour control. The reason for this is presumably that the oxygen contribution from an individual vessel tree becomes greater, due to higher oxygen gradients. This means that when the trees are isolated and diffusion is calculated, the apparent vessel density becomes low, causing more oxygen to leave the vessels, due to the poor oxygenation in the surroundings. This, in turn, causes the oxygen to transport further from the vessel, giving a larger fraction of oxygenated tissue when the sub-fields of different trees are combined. Therefore, the result of the ITM was misleading and the agreement with CTM was poor. The ITM also fails to capture obvious correlations (Fig 8) and is, consequently, clearly inadequate for its purpose, as is the CTM, when applied only to sub-volumes of the tumour, because of the lack of periodicity in the vessel structure. The results from the KS tests clearly show that the intra-tumoural heterogeneity on a larger scale is too pronounced for sub-samples on the order of 10^7^ voxels, side of 10 μm, to give a good estimate of oxygen distribution. At higher vascular densities, the spatial variations in oxygenation are likely to decrease, but as tumours generally have irregular vascularisation and oxygenation,[25] sub-sampling is preferably avoided.

The tumour model used here is easy to modify regarding branching characteristics, vessel oxygen levels, vessel density, vessel size, number of generations, number of trees etc. The cut-off in capillary length, which essentially means that capillaries shorter than 40 μm are excluded, is reasonable. This is due to that these vessels in general would run very close to the parent vessels, in fact partly overlap in most cases, given the branching angles and resolution. As a consequence of the use of constant vessel oxygen content, their contribution to the oxygen pressure field would most likely be completely negligible and including them would simply weigh the model down, although it can be done if desired, but preferably in combination with an increase in resolution.

The correlations between pO_2_ distribution characteristics and vascular fraction imply that parametric models may be constructed for, presumably faster, image based oxygenation estimations, given that the circumstances (e.g. variable relations and computational load) allow it. Initial efforts at this have been made.[5] There is likely to exist an optimal microvessel density resolution, for which the correlation is maximised and, from what we see here regarding interactions between adjacent vessel trees, this optimal resolution may vary with the structure of the microvascular network. This assumption is further supported by the weaker correlation between vascular fraction and anoxia (HF_0.01_).

In this study, we show how stochastic methods may be used to model, with a realistic appearance and on a clinically relevant scale, the chaos that is tumour vasculature. The estimated simulation times for computing the oxygenation of the entire tumour makes it ineffective for repeated investigations of factors that affect the oxygen distribution in a macroscopic sized tumour, e.g. the deflection angles at the branching points, the variations in artery length and the oxygen level in the vessels. Figs 6 and 7 as well as Tables 2 and 3 and the KS test results indicate a notable variation in oxygen distribution within and between individual samples which makes the common model expansion by mirroring or repetition an inappropriate measure. To access the full potential of this type of modelling, evaluation therefore should be performed on a cluster of processors, preferably one or several CUDA-compatible Graphic cards (GPU) or equivalent, so that computations may be parallelised, thereby considerably reducing the computation time so that entire tumours may be studied using sufficient resolution.

## Supporting Information

S1 File Oxygen levels for sub volume 1 using ITM. 30x30x30 matrix containing the oxygen levels for sub volume 1. Data is single precision, little endian.
(BIN)

S2 File Oxygen levels for sub volume 2 using ITM. 30x30x30 matrix containing the oxygen levels for sub volume 3. Data is single precision, little endian.
(BIN)

S3 File Oxygen levels for sub volume 3 using ITM. 30x30x30 matrix containing the oxygen levels for sub volume 3. Data is single precision, little endian.
(BIN)

S4 File Oxygen levels for sub volume 4 using ITM. 30x30x30 matrix containing the oxygen levels for sub volume 4. Data is single precision, little endian.
(BIN)

S5 File Oxygen levels for sub volume 5 using ITM. 30x30x30 matrix containing the oxygen levels for sub volume 5. Data is single precision, little endian.
(BIN)

S6 File Oxygen levels for sub volume 1 using CTM. 300x300x300 matrix containing the oxygen levels for sub volume 1. Data is single precision, little endian.
(ZIP)

S7 File Oxygen levels for sub volume 2 using CTM. 300x300x300 matrix containing the oxygen levels for sub volume 3. Data is single precision, little endian.
(ZIP)

S8 File Oxygen levels for sub volume 3 using CTM. 300x300x300 matrix containing the oxygen levels for sub volume 3. Data is single precision, little endian.
(ZIP)

S9 File Oxygen levels for sub volume 4 using CTM. 300x300x300 matrix containing the oxygen levels for sub volume 4. Data is single precision, little endian.
(ZIP)

S10 File Oxygen levels for sub volume 5 using CTM. 300x300x300 matrix containing the oxygen levels for sub volume 5. Data is single precision, little endian.
(ZIP)

S11 File Vessel data for sub volume 1. 330x330x330 matrix containing the vessel representation for sub volume 1. Data is single precision, little endian.
(ZIP)

S12 File Vessel data for sub volume 2. 330x330x330 matrix containing the vessel representation for sub volume 3. Data is single precision, little endian.
(ZIP)

S13 File Vessel data for sub volume 3. 330x330x330 matrix containing the vessel representation for sub volume 3. Data is single precision, little endian.
(ZIP)

S14 File Vessel data for sub volume 4. 330x330x330 matrix containing the vessel representation for sub volume 4. Data is single precision, little endian.
(ZIP)

S15 File Vessel data for sub volume 5. 330x330x330 matrix containing the vessel representation for sub volume 5. Data is single precision, little endian.
(ZIP)
